# O091: Healthcare associated infections: an overview (videoclip)

**DOI:** 10.1186/2047-2994-2-S1-O91

**Published:** 2013-06-20

**Authors:** C Palos, A Bispo, E Noriega, A Arnaut, A Cabral, P Nobre

**Affiliations:** 1 Infection Control Committee, Portugal; 2 Hospital Beatriz Ângelo, Loures, Portugal; 3 Infection Control Committee, Hospital do Barlavento Algarvio, Portimão, Portugal; 4 Nurse Directorate, Espírito Santo Saúde Group, Hospital Residencial do Mar, Lisbon, Portugal; 5 ADVITA, Hospital Residencial do Mar, Lisbon, Portugal; 6 Nurse Infection Control, Hospital Residencial do Mar, Lisbon, Portugal

## Background

HAI prevention and control is an essential component of patient safety. Healthcare workers awareness, knowledge and adherence are crucial in order to obtain the best results. This videoclip aims to provide healthcare workers (physicians, nurses, etc) on general concepts and practices on infection control, using a combination of avatar technology and real persons. HAI prevention and control is presented as an universal and timeless challenge, mainly based on adoption of good practices of hand hygiene and protective individual equipment. The main components and objectives of HAI prevention and control are also presented. Videoclips are a part of a global awareness and teaching materials on Infection Control and Prevention, available on hospital intranet and on infection control committee sessions.

## Introduction

HAI prevention and control is an essential component of patient safety. Healthcare workers awareness, knowledge and adherence are crucial in order to obtain the best results.

## Objectives

Improve awareness and knowledge on HAI prevention and control.

## Methods

This videoclip aims to provide healthcare workers (physicians, nurses, etc) on general concepts and practices on infection control, using a combination of avatar technology and real persons. HAI prevention and control is presented as an universal and timeless challenge, mainly based on adoption of good practices of hand hygiene and protective individual equipment. The main components and objectives of HAI prevention and control are also presented. Videoclips are a part of a global awareness and teaching materials on Infection Control and Prevention, available on hospital intranet and on infection control committee sessions.

## Results

N/A

## Conclusion

N/A

## Disclosure of interest

None declared

